# Housework Division and Second-Child Fertility Anxiety among Couples in China: The Urban and Rural Differences

**DOI:** 10.3390/ijerph16203910

**Published:** 2019-10-15

**Authors:** Jingyue Zhang, Yipeng Tian

**Affiliations:** 1 Institute of Gender and Culture, Changchun Normal University, Changchun 130032, China; zhangjingyue@ccsfu.edu.cn or; 2 Department of Sociology, School of Philosophy and Sociology, Jilin University; Changchun 130012, China

**Keywords:** housework division, couples of childbearing ages, second-child fertility anxiety

## Abstract

This study investigated the moderating role of household registration in the relationship between housework division and second-child fertility anxiety among Chinese couples of childbearing ages. Multilevel cluster sampling was used to select 1834 respondents aged 20–45 years from Jilin Province in China between 2016 and 2017. A sample of 542 adults who were married and had only one child was included in the final analysis. Hierarchical multiple regression was used to examine the proposed hypothesis. The results showed that the association between housework division and second-child fertility anxiety was significant in rural areas. However, the above association was not significant in urban areas. Household registration status was found to have a moderating effect on the relationship between housework division and second-child fertility anxiety. Differences in gender and fertility ideology have led to different housework divisions in urban and rural areas, which in turn have led to different effects on the second-child fertility anxiety of couples of childbearing ages in these areas.

## 1. Introduction

The division of housework in families is an important part of the cooperation between couples [1]. At the same time, it is also a key indicator that reflects the gender relationship of the family. With the popularization of the gender equality ideology and the increase in women’s education levels, the rate of female labor participation in the public sphere is increasing, whereas the gap between men and women in housework division is still relatively large [2,3]. Though research has shown that men are performing more housework than ever [4,5], women’s housework hours have not significantly decreased [6]. The division of housework has a significant impact on family patterns, marital satisfaction, occupational differentiation, labor efficiency, personal mental health, and fertility rate, and it can directly affect fertility intention and behavior [7,8,9,10]. Studies have shown that if women’s expectation for the division of housework before marriage is inconsistent with the actual situation after marriage, the likelihood of having two children is decreased [11]. 

Different from Western countries, China’s typical hukou system has created a two-tier urban–rural system in China. Hukou refers to household registration status, which is generally based on an individual’s birthplace. Rural populations generally hold agricultural household registration status, and those living in urban areas hold non-agricultural household registration status. Individuals with different hukou status have different levels of access to education, employment, housing and social welfare services and benefits. After the implementation of the policy of reform and opening up (Chinese economic reforms in 1978 led to a significant transition of China’s economy from a planned economy model to a market-oriented economy), China’s urban–rural differences have become increasingly prominent [12]. The problem of urban–rural differences has manifested in not only economic and political aspects but also in social and cultural aspects. Influenced by the historical context of different eras, the socioeconomic development of different regions, and the background of social and cultural policies, people of different generations in different regions tend to have different gender and fertility ideologies [13].

In urban China, economic and scientific spheres are rapidly developing, and domestic labor market resources are sufficient. These factors have alleviated the burden of domestic work among women who live in urban areas. However, in rural China, living conditions are poorer, social service levels are limited, and domestic market resources are relatively scarce. Compared with urban women, rural women need to take on more housework [14]. Research has shown that contrasted with men, women’s gender equality ideology is more modern [15]. Especially in rural areas, the transformation of family structure has weakened the power of previous generations in traditional families, whereas most rural men still regard the family clan relationship as more important than a couple’s relationship, and their fertility ideology is more susceptible to the traditions of previous generations [16]. However, the increasing education level and germination of the subjective consciousness of rural women have encouraged them to participate more in public affairs. Thus, most rural women do not want to have a second child. The hysteresis of the fertility ideology of rural men has led to differences in fertility decisions with their wives, creating family conflict. Therefore, both men and women have fertility anxiety when faced with fertility ideology differences with their family members. Generally, the more housework a woman bears, the weaker her second-child fertility desire and the more prominent the family conflict, which can lead to fertility anxiety in couples.

Following its reform and opening up policy, China’s public and private sectors have gradually been divided, and the division of housework has always been regarded as a problem in the private sector without attracting sufficient attention. In fact, there is no clear boundary between the private and the public sector, and the two are always infiltrating each other. However, research on the division of housework in China is very limited. At present, related research has mainly focused on issues such as gender equality ideology, labor time allocation, and income distribution. Few studies have paid attention to the impact of housework division on fertility. Especially in the context of the urban–rural dual structure, the urban–rural differences of division of housework have differing effects on fertility intention in urban and rural populations of childbearing age, including varying influences on their mental health. This study explores the moderating role of household registration in the relationship between the division of housework and fertility anxiety. According the results of the model, we propose suggestions to improve the fertility anxiety of couples of childbearing ages.

### 1.1. Defining Second-Child Fertility Anxiety

Anxiety has a psychological definition. The original meaning of anxiety refers to a kind of emotional response of an individual facing uncertain danger and a lack of effective self-protection [17,18]. In contrast, sociologists believe that anxiety is not only a psychological concept but also a sociological concept that could reflect individuals’ social environment and can explain social phenomena [19,20,21,22].

To release the stress of the transformation of the population structure in China, in 2016, the Chinese government implemented its universal two-child policy. Every couple of childbearing age could have a second child. However, most couples did not respond to the policy by having a second child. In modern society, the cost of parenting a child is much higher than ever, and with the increase in women’s public labor participation, most families lack child care resources [23]. However, in China, fertility is not the sole domain of couples of childbearing ages; it is a province of extended families. Generally, previous generations held a relatively traditional fertility ideology, such as preferring sons, viewing more sons as a blessing, and seeking sons to ensure the continuity of the clan. Younger generations, limited by realistic conditions, often view themselves as unable to have a second child. In this situation, couples may face conflicts with the previous generations of the family [24]. At the same time in China, especially in rural areas, influenced by a culture of filial piety, most husbands regard their relationship with their parents as more important than the relationship with their wife, and their fertility intention is similar to that of their parents [16]. Therefore, when making decisions about having a second child, husbands and wives may experience conflicts. When these conflicts appear, both husbands and wives often experience anxiety, referred to as second-child fertility anxiety.

### 1.2. Housework Division

Housework refers to unpaid labor in the family setting, such as laundry, cleaning, food preparation, and other tasks necessary to maintain a “livable” home [3]. Housework is the main way to achieve family reproduction and maintain family relationships [25,26]. Housework division refers to the distribution of housework undertaken by family members, especially by husbands and wives [27]. Because housework is unpaid work, the mode of housework division can directly reflect the family members’ divisions, especially the husband’s and wife’s gender ideology, gender equality-related behavior, and equality of welfare [14,28].

Given the dramatic increase in women’s education level and paid labor force participation, there has been escalating sociological attention paid to gender differentials in unpaid housework division [29,30,31,32,33,34]. Mainstream theories include new family economics theory, time availability theory, and relative resource theory. New family economics theory was proposed by Gary Becker. He assumed that couples feature economically rational people and that in a family, compared with the member who earns higher income in the public sector, the member who earns lower income will bear more housework [29]. Time availability theory holds that housework division is symbolic of gender relations in a family. The distribution of labor time between husbands and wives in the public and private sectors directly reflects the gender ideology of a family [31,34,35]. Though couples’ gender-related attitude toward housework division is more equal than ever, substantial gender ideologies and the allocation of housework have experienced no significant change [36]. Relative resource theory emphasizes that housework division reflects the power between husbands and wives in families [32,37]. The increasing of education level and income could reduce housework time [26,38]. Accordingly, gender equality ideology, the allocation of relative resources, the validity of time in public and private sectors, and sociocultural context all influence housework division [26]. Finally, housework division can affect a couple’s mental health, including marriage satisfaction and subjective well-being [10,39].

In China, the transformation of the economic structure changed traditional gender ideology and promoted more equal housework division; however, the housework labor time of women is still much longer than that of men. Wives are still the main bearer of daily housework [28,40]. At the same time, due to differences in economic development and sociocultural contexts between urban and rural areas, housework division in urban and rural areas is different in China [14]. Compared with urban China, living conditions and social service levels in rural China are relatively poor, and the gender ideology in rural China is more traditional. Therefore, women in rural China undertake more housework than their counterparts in urban China. An excess of housework can produce negative effects on rural women’s income, competitiveness in the public sector, and their welfare level, as well as a lowering of family status among rural women, all of which lead to a vicious circle regarding rural women’s time allocation in housework and public labor sectors [14].

### 1.3. Housework Division and Fertility Anxiety

After World War II, the education level of women in Western countries increased rapidly. In the context of promoting productivity and the transformation of labor type, women got more opportunities to participate the public labor sector [41]. Thus, the opportunity costs of fertility became much higher than before. In other words, fertility behavior can affect the promotion of income and prospective earnings in the labor market among women [29]. At the same time, influenced by the transformation of women’s gender ideology and the promotion of women’s income and social status, women are expected to make fertility decisions on their own [42]. However, in the process of the transformation of gender equality ideology, men lag behind women [43]. To maintain a stable and harmonious relationship between husband and wife, most women chose to undertake more housework to balance the relationship between family and society, releasing their husband’s mental stress [28]. In this situation, considering the opportunity costs of fertility and the dual pressures of family and work, married professional women have to make an choice between fertility and self-actualization [44,45]. 

In China, before the implementation of the policy of reform and opening up, most enterprises were state-owned and had their own nurseries for their employees, which greatly eased the family burden of women who participated in the public labor sector. However, given the market economy reforms since late 1970s, enterprises began to seek maximization of profits. In order to save production costs, enterprises gradually reduced the subsidies and benefits in terms of family reproduction [43]. Furthermore, influenced by traditional gender ideology, these functions of family reproduction that have returned to the family have been mainly borne by women [46,47]. The dual pressures of public–private responsibilities have had a significant impact on Chinese women’s fertility intentions [43], which has further influenced their fertility behaviors. In the context of traditional Chinese culture, they may have conflicts with their husbands or previous generations, creating anxiety about whether to have a second child [42].

### 1.4. Role of Household Registration in Housework Division and Fertility Anxiety

In China, traditional culture significantly influences people’s fertility ideology, and different generations have different fertility ideologies [1]. In recent decades, family size in China has continually decreased, and the traditional extended family structure has been replaced by the modern core-family structure, along with the rapid transformation of women’s social and family status, fertility ideology, and fertility behavior [6,47,48]. Influenced by the popularization of gender equality, in urban China, more women can make decisions on their own [14]. However, in rural China, although many couples also build a core family and live apart from previous generations, husbands often regard their relationship with their parents as more important than their relationship with their wife [16]. Therefore, in rural China, relationships among generations and within couples likely influence fertility intention [43]. At the same time, compared with their counterparts in urban areas, women in rural areas undertake more housework, and the stress of doing housework and germinating self-awareness often prompt them to have fewer children [1,49,50]. In rural China, the fertility ideology of previous generations is more traditional than urban China [51]. Thus, when facing conflicts with their parents or parents-in-law, women in rural areas frequently experience more anxious feelings about whether to have a second child [42,52]. Because men in rural China face dual pressure from the conflicts between previous generations and their wife, they also experience second-child fertility anxiety [42].

Accordingly, we posit the following hypotheses: Housework division influences second-child fertility anxiety. Household registration has a moderating effect on the relationship between housework division and second-child fertility anxiety. The effects of housework division on second-child fertility anxiety are more significant in rural China.

## 2. Materials and Methods

### 2.1. Sampling

The data for this study were derived from a survey named The Influence of the Universal Two-Child Policy on Female Fertility Behavior conducted in Jilin Province by the Institute of Gender and Culture at Changchun Normal University. A secondary data analysis was used in this study. The survey occurred from October 2016 to April 2017 in eight cities. Jilin Province is in Northeast China, where the main financial resource is agriculture. Thus, in the rural areas of Jilin Province, most women need to do not only housework but also some agricultural work. Compared to their counterparts in urban areas, the labor burden of women in rural areas is much heavier. In addition, because women in rural areas have few opportunities to participate in public labor, they have no independent income, leading to low family status. They often must do extensive housework and take care of their children. They frequently do not want to have a second child to add their work burden; however, they have less power in their marital and family relationships. These factors can produce conflicts in the family and lead to second-child fertility anxiety. Data collection involving human respondents was conducted according to the ethical standards of the institutional research committee and in accordance with the Helsinki declaration. Oral consent was obtained from all the respondents. Ethics approval was obtained from the Institute of Gender and Culture, Changchun Normal University.

The survey used multilevel cluster sampling as the sampling method. First, the researchers calculated the relative population of the eight cities according to the population proportions of the local representative samples from the sixth national consensus. According the proportions, sample size was calculated and allocated in the eight cities. The inclusion and exclusion criteria are as follows: Eligible respondents needed to (a) be between 20 and 45 years of age (through October 2016); (b) be married; (c) have at least one child. The respondents needed to have adequate cognitive function and listening capacities to complete the survey. Of 1920 questionnaires distributed, 1834 valid questionnaires were returned. More than 80% of questionnaires were valid. The survey assessed the respondents’ basic situation, fertility intentions, effects of the universal two-child policy on their work and family lives, and factors affecting their decision to have one more child. The survey used four options to measure fertility intentions: “I want to have a second child,” “I am not sure,” “I am thinking about it,” and “I do not want to have a second child.” Respondents who chose the first and last answers indicated that they had confirmed their fertility decision—whether they did or did not want to have a second child, they were unlikely to have second-child anxiety. In contrast, those who chose “I am not sure” or “I am thinking about it” would likely have second-child fertility anxiety, because they have some second-child fertility intention and have not made a final determination. Accordingly, we selected the participants who chose the second and third answers—i.e., 542 individuals. We calculated sample size based on the average effect size of 0.20 reported in the literature [42]. For 80% power, a two-tailed α-level of 0.05, 20 predictors, and 122 respondents are needed to detect the effect [53]. Research data are available in the Supplementary Materials, File S1.

### 2.2. Measurements

#### 2.2.1. Dependent Variable: Second-Child Fertility Anxiety

First, we used a question—“After the implementation of the universal two-child policy, do you want to have a second child?”—to screen the participants. As previously described, we selected respondents who chose the answers “I am not sure” and “I am thinking about it.” Then, we used another question to measure these respondents’ second-child fertility anxiety: “Imagining that someone else has two children and you do not have the ability to have a second child, tell me the degree of anxiety you feel.” The variable was measured on a 5-point Likert scale (1 = Not anxious at all, 5 = Very anxious).

#### 2.2.2. Independent Variable: Housework Division

We used one survey question to measure this variable: “What is the mode of the housework (included cleaning, washing, preparing food and take care of children) division in your family? How much housework does the wife in your family need to undertake?” Participants had three answer options: (a) The wife does not to undertake the housework at all; (b) the wife undertakes about half of the housework; and (c) the wife has to undertake all of the housework. We coded the answers as 1–3, respectively; thus, higher scores indicated that the wife undertook more housework in the family.

#### 2.2.3. Moderator Variable: Household Registration

We coded household registration as a dichotomous variable. In China, household registration is mainly divided into two categories: Agricultural (i.e., rural) and non-agricultural (i.e., urban) household registration. Agricultural household registration was coded as 0, and non-agricultural household registration was coded as 1.

#### 2.2.4. Control Variables

Control variables included age, gender, education level, type of occupation, family per capita income, parenting costs, fertility ideology, the husband’s status as the only son, and the gender of the first child. Age was calculated by year of birth. Male was coded as 1, and female was coded as 2. Four categories were used to indicate the education level of the respondents, coded as 1 (primary school and below), 2 (junior high school), 3 (high school), and 4 (college and above). The type of occupation was divided into two categories: “In-system work” was coded as 1, and “out-system work” was coded as 2. The former included civil servants, workers in public institutions, and workers in state-owned enterprise; the latter included farmers, employees of private and foreign companies, and freelancers. We divided family per capita income into six levels, from 1 (less than 1000 yuan) to 6 (greater than 5000 yuan). Parenting costs were measured as direct and indirect parenting costs. Three questions were used to measure direct costs: (a) Do you think having a second child will reduce your family life quality? (b) Do you think having a second child will have negative impact on your family’s economic situation? (c) Do you think having a second child will reduce your personal life quality? We used “yes” (coded as 1) and “no” (coded as 0) as the answer options. A summed score for the three questions was used. Higher scores indicated greater direct parenting costs. To measure indirect parenting costs, we used two questions. First, “How do you think having a second child will affect your career?” Participants had four answer options: Affecting personal career development, affecting economic income, affecting individual career advancement, and reducing opportunities for individual re-education and training. The more answers the respondents chose, the greater impact on their occupation. The other question was, “Do you think that having a second child will reduce your entertainment time and space?” The alternative answers were “yes” (coded as 1) and “no” (coded as 0). We added the scores of the two questions; higher scores reflected greater indirect costs. Another question was used to measure fertility ideology: “The following options are about fertility ideology, which ones do you agree with?” Answers representing traditional fertility ideology were: “The more sons, the more blessing;” “Bring up sons to provide for one in old age;” “Having a child could carry on the family line;” and “Fertility is the value of a women.” Answers representing modern fertility ideology were: “The aim of fertility is participating in the process of a child growing up” and “Fertility is a method to promote the relationship between husband and wife.” Each affirmative response resulted in one point, and we used the score of modern fertility ideology minus the score of traditional fertility ideology to measure respondents’ fertility ideology, because the scores we calculated included negative number and zero (the scope of the score was from –4 to 3); for convenience, we recoded the scope of the score from 1 to 8. We determined whether the husband was the only son in his family, measured as “yes” (coded as 1) or “no” (coded as 0). Finally, the gender of the couple’s first child was coded as 1 for male and 2 for female.

### 2.3. Data Analysis

We used a hierarchical regression analysis to test the moderating role of household registration in the relationship between housework division and second-child fertility anxiety. Hierarchical regression analysis was conducted in three steps. First, the sociodemographic characteristics and socioeconomic status of the respondents, the gender of the first child, and household registration status were entered in the regression model. Second, second-child fertility anxiety was regressed on housework division. Third, a two-way interaction term of household registration and housework division was entered in the model to test whether the association between housework division and second-child fertility anxiety varied by household registration. Regression coefficients for housework division were calculated by conducting two separate regression models for those with different household registration status. A data analysis was conducted using SPSS 22.0 (SPSS Inc., Chicago, IL, USA). 

## 3. Results

### 3.1. Descriptive Statistics

First, a descriptive analysis of all variables was performed (Table 1) using distribution frequency and valid percentages for nominal variables and means (M) and standard deviations (SD) for continuous variables. We also reported the score range of the variables. The score range of second-child fertility anxiety was from 1 to 5, with a mean of 2.02 (SD = 1.07). In this sample, 18.3% of wives did not undertake housework, 60.9% performed half of the housework, and 20.8% undertook all of the housework. The distribution of household registration was relatively equitable, with 54.1% of urban household registration status and 45.9% of rural household registration status. The respondents ranged in age from 20 to 45 (M = 34.97, SD = 5.02). The sample featured 272 men and 270 women. Most respondents had a high school education, accounting for 41.9% of the sample. The proportion of occupation type was 42.1% in-system workers and 57.9% out-system workers. Regarding family per capita income, 28.0% of the respondents reported receiving 3001–5000 Yuan. The score scope of direct parenting costs was 0–3, with a mean of 1.85 (SD = 0.99), and that of indirect costs was 0–5, with a mean of 1.69 (SD = 1.40). The mean fertility ideology was 5.02 (SD = 1.38). Nearly half (44.5%) of the respondents or their husbands were an only son, and 50.4% of the respondents’ first child were male. An independent t-test was conducted to test differences among all variables in the two household registration groups. We found no significant differences in all variables except second-child fertility anxiety between the two groups.

### 3.2. Module Results

We constructed three models to analyze the data (Table 2). First, we used variance inflation factor (VIF) and tolerance values to test the issue of multicollinearity, and we found no evidence for multicollinearity (VIF < 10, tolerance values > 0.2). In the first model, we added control variables (R^2^ = 0.198, Durbin–Watson statistic = 1.889). In the second model, we added the variable of housework division (R^2^ = 0.199, Durbin–Watson statistic = 1.882), and the model did not change significantly. We found that housework division had no statistically significant effects on second-child fertility anxiety (B = 0.065, t = 0.956, *p* > 0.05). According to gender equality theory [6,43], housework division should influence second-child fertility anxiety; thus, we considered the moderating role of household registration. In the third model, we added the interaction terms of household registration and housework division, and the model changed significantly (R^2^ = 0.205, Durbin–Watson statistic = 1.906). We found household registration had a moderating effect on the relationship between housework division and second-child fertility anxiety (B = −0.282, t = −2.046, *p* < 0.05). Therefore, we concluded that the moderating effect of household registration offset the influence of housework division on second-child fertility anxiety.

### 3.3. Moderation Effect of Household Registration

**Figure 1:**
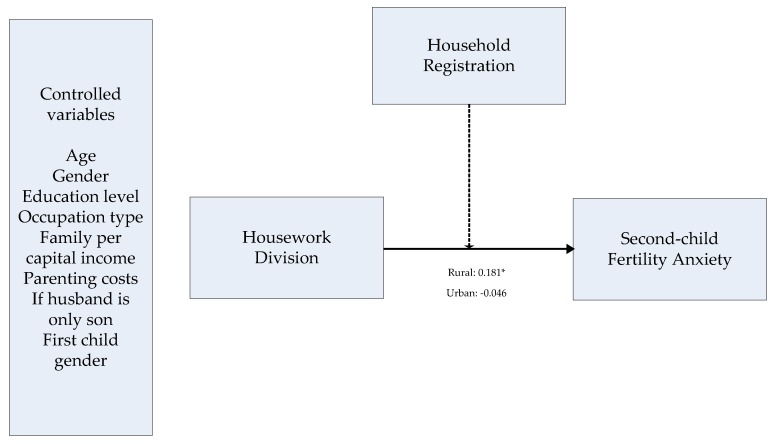
Model of the role of household registration in the relationship between housework division capital and second-child fertility anxiety. Notes: Standardized coefficients are reported. The dashed line indicates a moderating effect. * *p* < 0.05 (two-tailed).

After exploring the moderating role of household registration, we tested the relationship between housework division and second-child fertility anxiety in urban and rural groups through a hierarchical regression analysis (Figure 1). The statistical test values are as follows: For the urban group, R^2^ = 0.241 and Durbin–Watson statistic = 1.917; for the rural group, R^2^ = 0.186 and Durbin–Watson statistic = 1.872. The results of the two models further verified the moderating role of household registration on the relationship between housework division and second-child fertility anxiety. Household division was positively associated with fertility anxiety only among rural couples (B = 0.181, t = 2.034, *p* < 0.05).

## 4. Discussion

This study analyzed the influence of housework division on second-child fertility anxiety in couples of childbearing ages and further analyzed the moderating role of household registration. The results show that in urban areas, housework division had no significant impact on second-child fertility anxiety, whereas in rural areas, housework division significantly influenced second-child fertility anxiety.

Fertility intention and fertility behavior are two different concepts; fertility intention influences fertility behavior through some mediators, such as fertility attitude, fertility decision, and fertility anxiety [54]. The majority of the literature has focused on fertility behavior and its social determinants. A few studies have suggested that housework division plays an important role in influencing fertility behaviors among Chinese families [39,49,50]. However, there is a lack of studies focusing on housework division and second-child fertility anxiety. The present study adds new empirical evidence to the literature by examining the above association. The findings of this study provide a base for future studies to further examine the mechanisms linking housework division, fertility anxiety, and fertility behavior (e.g., fertility anxiety as a mediator). Given its decreasing fertility rates, China will encounter a number of demographic changes, including but not limited to the shortage of population in labor ages, an imbalance of the sex ratio, and population aging. Under such circumstances, the Chinese government recently implemented the “universal two child” policy. However, the majority of couples of childbearing ages do not choose to have a second child. Many adults of childbearing ages have second-child fertility anxiety. Understanding the relationship between housework division and second-child fertility could help us improve fertility related policies and increase the fertility rate in China. 

In urban China, most people have accepted the gender equality ideology; thus, most couples in urban areas tend to embrace a relatively equal mode of housework division [14]. However, in rural China, limited by the lower education level of women and the influence of the traditional cultural preference for men, the acceptance level of gender equality ideology for rural women is just beginning to increase [55]. At the same time, the acceptance level of gender equality ideology among men is lagging behind that of women, and most rural men have not accepted the gender equality ideology [13,31]. Thus, in rural areas, women are still the main bearers of housework [50]. Second, the mechanization of housework and housework services resources for affluent individuals in urban areas vastly reduces women’s housework-related stress [1]. In rural areas, however, the mechanization of housework is not developed and housework services resources are lacking, forcing women to do most of the housework [14]. Third, compared with women in urban areas, the education level of rural women is lower, they have fewer opportunities to participate in public work, their family and social status are lower, and they have fewer autonomous rights in the family, so they have to undertake more housework. Finally, in rural areas, most couples live near their parents, and their housework often includes taking care of their parents and parents-in-law, which can aggravate their labor burden. In urban areas, most couples live in core families, and their parents have a pension to take care of themselves [28]. Thus, the housework labor burden for urban women is much less than their counterparts in rural areas. In conclusion, rural women have to undertake much more housework than women in urban areas, and given their heavy housework burden, women in rural areas often do not want to have a second child. However, their low family status, lack of autonomy in the family, and pressure from previous generations often make them more anxious regarding whether to have a second child. At the same time, facing dual pressure from previous generations and their wives, husbands in rural areas also have second-child fertility anxiety.

Some limitations exist in this study. First, the data used in the study were cross-sectional. Thus, we could not determine a clear causal relationship between the dependent variable and independent variable. However, the findings of the study are supported by theories, and the independent variable influenced the dependent variable at a statistically significant level, indicating that we can accept the conclusions of the study. Second, the data were derived from a survey in Jilin Province, China. The economic development, social development, and cultural context of Jilin Province differ from many other regions in China, excluding provinces in Northeast China and some western provinces. Therefore, the conclusions of the study are likely representative of Jilin Province and some other regions with similar economic and social development, cultural contexts, and population structure. These results might not be generalizable to regions that are significantly different than Jilin Province. In future research, longitudinal national data should be used to analyze related issues, such as housework division, gender equality, and fertility-related issues. Finally, conditions related to fertility anxiety might be different for women and men in the Chinese contexts, which need further explorations in future qualitative studies. 

## 5. Conclusions

The findings suggested that in the whole model, housework division had no significant influence on second child fertility anxiety. This is because the moderating effect of household registration offset the influence. For further explore the moderating role of household registration in the relationship between housework division and second-child fertility anxiety according to related theories, we added the interaction variable of housework division by setting into the model and found the moderating role of household registration. Finally, we constructed regression on rural and urban groups separately. In the rural group, housework had a significant effect on second child fertility anxiety. In the urban group, however, housework had no significant effect on second child fertility anxiety. Policy makers and intervention designers should publicize gender equality ideology, especially in rural China, to promote the equalization of housework division and to increase the rate of male participation in housework. In this way, couples could reduce conflicts about fertility. In addition, females should fight for the right to speak and make fertility decisions based on their own intentions, and husbands should respect their wives’ rights to reduce conflicts about fertility and relieve second child fertility anxiety.

## Figures and Tables

**Table 1:** Sample characteristics (*N* = 542).

Variables	Frequency	Percentage	Mean (SD)
Fertility Anxiety			2.02 (1.07)
Housework Division (The Part Wife Undertakes)			
No at all	99	18.3	
About Half	330	60.9	
All	113	20.8	
Household Registration			
Urban	293	54.1	
Rural	249	45.9	
Age			34.97 (5.02)
Gender			
Male	272	50.2	
Female	270	49.8	
Education Level			
Primary School	60	11.4	
Junior School	171	32.5	
High School	226	41.9	
Collage and Above	75	14.2	
Occupation Type			
In-System	228	42.1	
Out-System	314	57.9	
Family Per Capita Income (Yuan)			
<1000	85	16.4	
1001–2000	81	15.3	
2001–3000	110	20.7	
3001–5000	152	28.0	
>5000	104	19.6	
Direct Costs			1.85 (0.99)
Indirect Costs			1.69 (1.40)
Fertility Ideology			5.02 (1.38)
If Husband Is Only Son			
Yes	241	44.5	
No	301	55.5	
First Child Gender			
Male	273	50.4	
Female	268	49.4	

**Table 2:** Multiple regression of housework division and second-child fertility anxiety.

Variables	1			2			3		
	B	SE	β	B	SE	β	B	SE	β
(Constant)	−1.341	0.466		−1.465	0.484		−1.730	0.499	
Age	0.023	0.009	0.110 **	0.023	0.009	0.108 **	0.025	0.009	0.116 **
Gender	0.234	0.084	0.110 ***	0.239	0.084	0.112 ***	0.249	0.084	0.117 ***
Household Registration	0.225	0.103	0.105 **	0.228	0.103	0.106 **	0.811	0.303	0.379 ***
Education Level	0.049	0.062	0.040	0.047	0.062	0.038	0.049	0.062	0.040
Occupation Type	0.167	0.112	0.077	0.173	0.112	0.080	0.155	0.112	0.072
Family Per Capital Income	0.079	0.039	0.105 **	0.078	0.039	0.104 **	0.069	0.039	0.091
Direct Costs	0.272	0.043	0.253 ***	0.271	0.043	0.253 ***	0.264	0.043	0.246 ***
Indirect Costs	0.097	0.039	0.126 **	0.101	0.039	0.132 **	0.098	0.039	0.128 **
Fertility Ideology	0.019	0.032	0.025	0.018	0.032	0.024	0.018	0.031	0.023
If Husband is Only Son	0.171	0.094	0.079	0.168	0.094	0.078	0.186	0.094	0.087 **
First Child Gender	0.272	0.084	0.127 ***	0.270	0.084	0.127 ***	0.244	0.085	0.114 ***
Housework Division				0.065	0.068	0.038	0.201	0.095	0.118 **
Housework Division × Rural Household Registration							−0.282	0.138	−0.290 **

Notes: *** *p* < 0.01, ** *p* < 0.05 (two-tailed).

## Supplementary Materials

The following are available online at https://www.mdpi.com/1660-4601/16/20/3910/s1, File S1: Research Data.
